# Comparison of a portable, pneumotach flow-sensor–based spirometer (Spirofy™) with the vitalograph alpha Touch™ spirometer in evaluating lung function in healthy individuals, asthmatics, and COPD patients—a randomized, crossover study

**DOI:** 10.1186/s12890-024-02972-4

**Published:** 2024-05-10

**Authors:** Deepak Talwar, S Balamurugan, Mahavir Modi, Sundeep Salvi, Meena Lopez, Rushika Shah, Abhijit Vaidya, Monica Barne, Sapna Madas, Nandan Kulkarni, Sandesh Sawant, Jaideep Gogtay

**Affiliations:** 1 Department of Pulmonology, Pulmonary Sleep & Critical Care, Metro Centre for Respiratory Diseases, WASOG Sarcoid Clinic, Noida, Uttar Pradesh India; 2 Department of Pulmonology, Chest & Diabetes Research Institute, Chennai, Tamil Nadu India; 3 Department of Pulmonology, Ruby Hall Clinic, Pune, Mumbai, Maharashtra India; 4 Pulmocare Research & Education Foundation (PURE), Pune, Maharashtra India; 5 Department of Medical Affairs, Cipla Ltd, Mumbai, Maharashtra India; 6 Training Research Programs, Chest Research & Training Pvt Ltd, Pune, Maharashtra India; 7 Department of Data Management & Statistics, Chest Research & Training Pvt Ltd, Pune, Maharashtra India; 8 CiplaMedTech, Cipla Ltd, Mumbai, Maharashtra India

**Keywords:** Asthma, Chronic obstructive pulmonary disease, Forced expiratory volume, Spirometer, Forced vital capacity

## Abstract

**Background:**

Spirofy™ is India’s first portable, pneumotach flow-sensor-based digital spirometer developed to diagnose asthma and chronic obstructive pulmonary disease (COPD). In this study, we compared the performance of the Spirofy™ device with that of the Vitalograph Alpha Touch™ spirometer in measuring the lung capacities of healthy individuals, asthmatics, and COPD patients. We also assessed the inter-device variability between two Spirofy™ devices.

**Methods:**

In a randomized, three-way crossover, open-label study, we measured the differences in forced expiratory volume in the first second (FEV_1_) and forced vital capacity (FVC) between the Spirofy™ and Vitalograph Alpha Touch™ spirometers. A proportion of the FEV_1_/FVC ratio distribution of < 0.7 was used to compare the diagnostic accuracies of the Spirofy™ with Vitalograph™ Alpha Touch™ spirometers.

**Results:**

Ninety subjects participated in this study. The mean ± SD FVC values obtained from the Spirofy™ 1, Spirofy™ 2, and Vitalograph Alpha Touch™ devices were 2.60 ± 1.05 L, 2.64 ± 1.04 L, and 2.67 ± 1.04 L, respectively. The mean ± SD FEV_1_ values obtained from the Spirofy™ 1, Spirofy™ 2, and Vitalograph Alpha Touch™ devices were 1.87 ± 0.92 (L), 1.88 ± 0.92 (L), and 1.93 ± 0.93 (L), respectively. A significant positive correlation was found between the FVC and FEV_1_ values recorded by Vitalograph Alpha Touch™, Spirofy™ 1, and Spirofy™ 2. As compared to Vitalograph Alpha Touch™, the Spirofy™ device showed good sensitivity (97%), specificity (90%), and overall accuracy (93.3%) at an FEV_1_/FVC ratio < 0.7. No inter-device variability was observed between the two Spirofy™ devices.

**Conclusion:**

Spirofy™ is a portable and easy-to-use device and is as accurate as the standard Vitalograph Alpha Touch™ spirometer for the diagnosis of COPD and asthma.

**Trial registration:**

CTRI/2021/09/036492 (Clinical Trials Registry - India)

**Supplementary Information:**

The online version contains supplementary material available at 10.1186/s12890-024-02972-4.

## Introduction

Globally, chronic respiratory diseases are the most common causes of morbidity and mortality [1] and lead to significant healthcare expenses [2]. Obstructive airway diseases (OAD), which include chronic obstructive pulmonary disease (COPD) and asthma, are a subset of chronic respiratory diseases that share common features [1]. Globally, asthma is the 16th cause of years-lived-with-disability and the 28th most common source of burden of disease [3]. In 2019, COPD was the 6th major cause of years-lived-with-disability for all ages and the third leading cause of death [4]. There are an estimated 50 million COPD cases and 34 million asthma cases in India and COPD is the second leading cause of death and disability-adjusted life years in India [5–7].

The gold standard test for the diagnosis of obstructive airway diseases is spirometry [8]. It is primarily used in patients with asthma and COPD [9]. It is a safe, practical, and reproducible test to evaluate lung function in primary care settings [10]. Under-use of spirometry is the most important cause of under-diagnosis of asthma and COPD; this occurs because of several barriers associated with its use in the primary care setup, viz., high cost, challenges in performing the test, and the extended time required to perform the test. It is believed that specialized training in performing the spirometry procedure and interpreting its results as well as improving the affordability and accessibility of the device could enhance its use [11, 12].

Cipla Limited has developed a new, digital point-of-care spirometer (Spirofy™), which is portable, easy to use, and based on pneumotach flow-sensing technology. Spirofy™ has undergone laboratory validation under different testing conditions. A randomized, open-label, three-way, crossover study was conducted to validate the performances of two Spirofy™ devices (Cipla, pneumotach sensor-based spirometer). The main objective of this study was to compare the performances of two Spirofy™ devices (device 1 and 2) with that of the Vitalograph Alpha Touch™ spirometer in measuring the lung capacities of healthy individuals, asthmatics, and patients with COPD. The secondary objective of this study was to assess the inter-device variability between the Spirofy™ devices.

## Methodology

### Study design

In this randomized, open-label, three-way crossover study, we compared the performance of two Spirofy™ devices to that of the reference Vitalograph Alpha Touch™ standard spirometer (pneumotach sensor-based spirometer) in measuring the lung capacities of healthy subjects, asthmatics, and COPD patients. Vitalograph Alpha Touch™ was chosen as the reference standard for evaluating Spirofy because it is widely used and is based on pneumotach technology, which is also utilized in the Spirofy™ device. The study was conducted over a period spanning from October 2021 to February 2022.

### Study population

The study population was comprised of randomly selected healthy subjects, asthmatics, and COPD patients visiting three respiratory clinics in India. The Independent Ethics Committee/Institutional Review Board (IEC/IRB) approved the informed consent, study protocol, and subject information sheets before the initiation of the study. A total of 90 subjects were included, comprising 29 healthy individuals, 31 asthmatics, and 30 COPD patients. The study was done as per the approved protocol requirement. The study protocol can be made available on request with justification. Requests for access should be directed to the corresponding author.

### Sample size

The sample size was calculated assuming an intra-subject standard deviation of 150 ml with a mean difference of 45 ml for FEV_1_ measured by 2 spirometers, with a power of 80% and 5% level of significance. Based on the calculations, a total of 90 subjects were required.

### Inclusion criteria

Healthy subjects, stable asthma patients, or stable COPD patients aged ≥ 18 years with medical history/records showing evidence of diagnosed asthma or COPD, no history of disease exacerbations in the preceding 4 weeks, and no other active or chronic respiratory complaints were included in the study. The other criteria for inclusion for healthy individuals were a declaration of normalcy after a clinical examination and a negative history of respiratory tract infection in the previous 4 weeks. The subjects had to test negative for the severe acute respiratory syndrome coronavirus (SARS-CoV-2) within the last 24–48 h or as per the respiratory clinic’s policy.

### Exclusion criteria

Patients with lung/airway diseases other than asthma or COPD were excluded from the study. Patients with (a) contraindications for spirometry as per the American Thoracic Society/European Respiratory Society (ATS/ERS) 2019 guidelines; (b) any significant deformity of the thorax or vertebral column that may cause persistent airflow limitations or altered lung volumes; (c) uncontrolled or unstable asthma/COPD status; and (d) severe concomitant diseases such as cardiovascular, pulmonary, abdominal, neurological, or endocrine disorders were also excluded. Pregnant women were also excluded from this study.

### Study devices

**Fig. 1:**
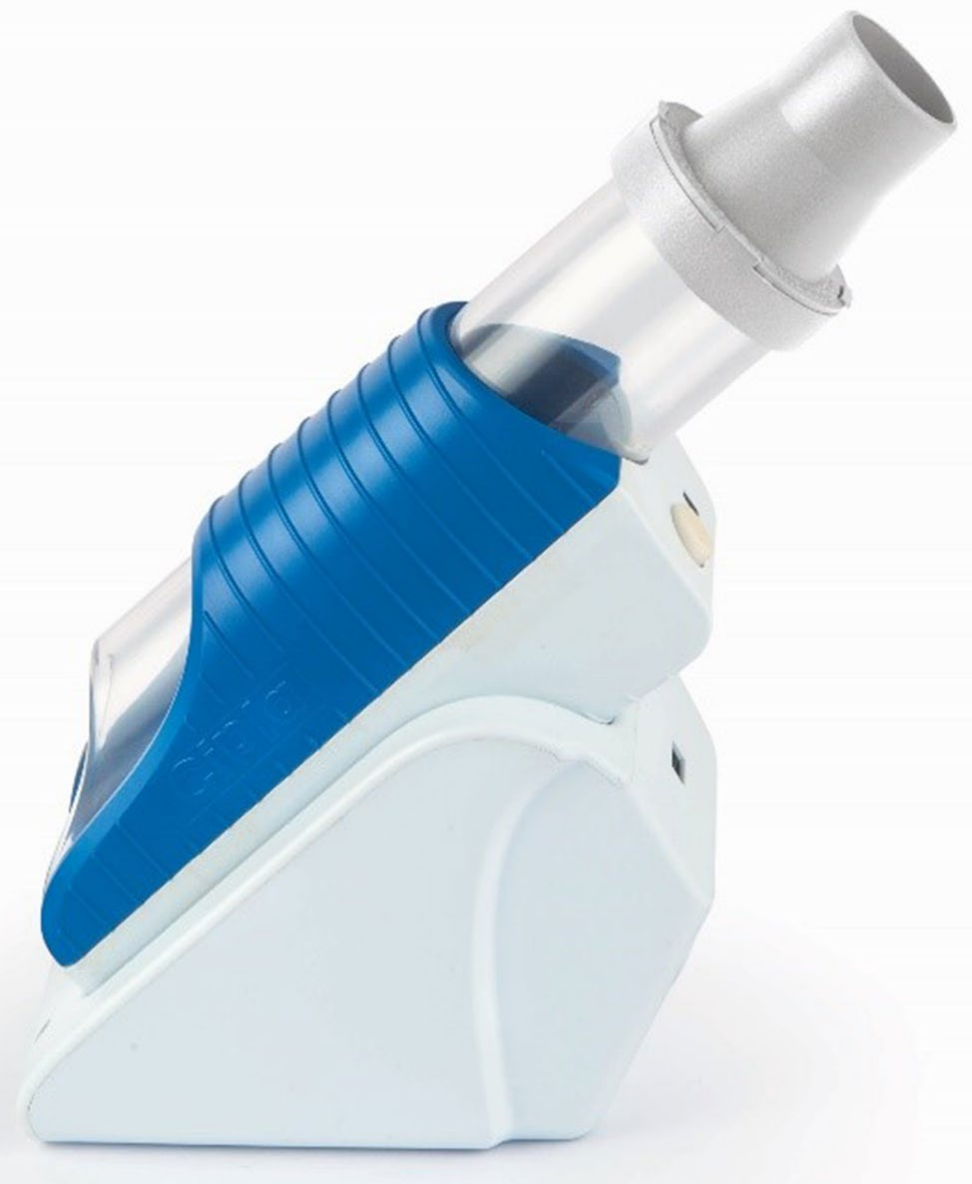
Image of Spirofy™ pneumotach sensor-based spirometer used in the study

**Fig. 2:**
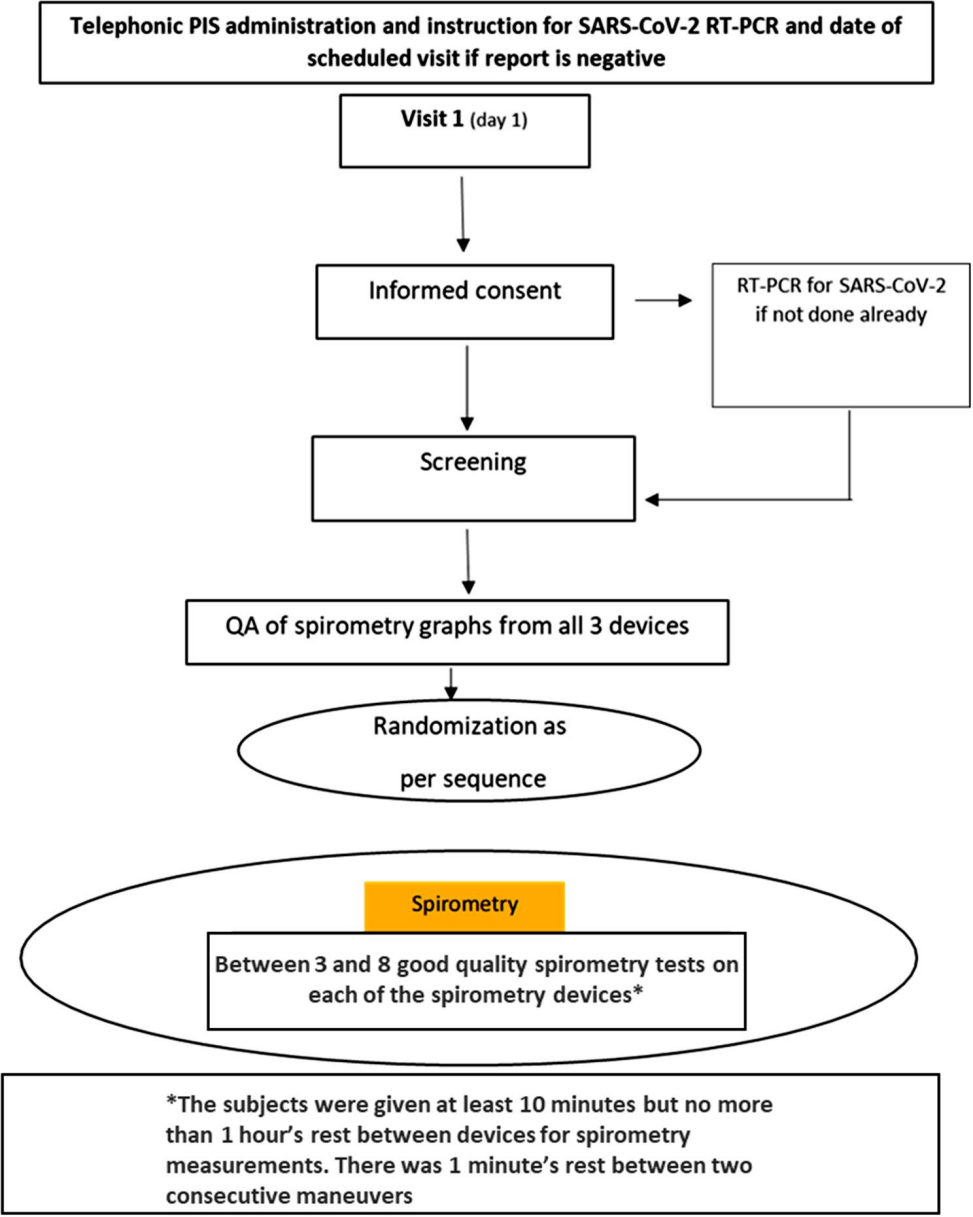
The schematic representing the study protocol. PIS: Patient information sheet; QA: Quality assurance; RT-PCR: Real-time-polymerase chain reaction; SARS-CoV-2: Severe acute respiratory syndrome coronavirus 2

The devices used in the study were: (1) Two Spirofy™ pneumotach sensor-based spirometers (Spirofy™ 1 and 2) manufactured by Cipla Ltd., India as test medical devices (Figs. 1) and (2) a Vitalograph Alpha Touch™ spirometer (pneumotach sensor-based spirometer) manufactured by Vitalograph Inc., USA as a standard for comparison.

### Screening and spirometry

Each subject underwent one screening and one study visit. Both visits were conducted on the same day on which the subject tested negative for the SARS-CoV-2 test or within 24–48 h (or as per site policy) of the negative result and only if the subject was willing to participate in the study. After the screening, informed consent was obtained, and the demographic characteristics and routine history of the individual were recorded (Fig. 2).

Three spirometry tests were performed on the participants (one test each with the two Spirofy™ spirometers of Cipla Ltd. and one with the Vitalograph Alpha Touch™ spirometer) in a pre-determined, randomized sequence generated for each of the three devices. Due to the random allocations of the subjects to all three device sequences, the study provided unbiased estimates for the differences in measured values between the devices. The cross-over design of the study allowed each subject to serve as his/her control to minimize the influence of potential confounders such as inter-subject variability. Details regarding clinical information and results from index/reference standard tests were available to the personnel administering the tests.

Spirometry was performed according to the ATS/ERS 2019 guidelines [13]. The study protocol included specific measures to control for the use of bronchodilators among participants on the day of spirometry testing. Prior to conducting spirometry, all participants were instructed to withhold any ongoing respiratory medication for a period consistent with the defined washout periods recommended by the ATS/ERS 2019 guidelines. Three to eight spirometry maneuvers were recorded with each spirometer to ensure that the subject had performed three acceptable and two reproducible maneuvers. Participants were requested to rest for at least 10 min between two spirometry tests, with the rest period not exceeding 1 h. Three sets of spirometry reports were generated for each subject on the same day during the same session of the day. The highest values obtained by the participants from each of the three acceptable and reproducible spirometry reports were used for further analysis.

### Primary endpoints

The primary endpoints were the differences in forced vital capacity (ΔFVC) and the differences in forced expiratory volume in the first second (ΔFEV_1_) between the Spirofy™ and Vitalograph Alpha Touch™ spirometers. The co-primary endpoint was the difference in FEV_1_/FVC ratio of < 0.7 between the Spirofy™ and Vitalograph Alpha Touch™ spirometers. The distribution of the FEV_1_/FVC ratio < 0.7 was used to assess the diagnostic accuracies of the Spirofy™ devices by comparing their performance with that of the Vitalograph in being able to differentiate subjects with OAD from healthy individuals; the results have been presented as the sensitivity, specificity, and overall accuracy of each device.

### Secondary endpoints

The secondary endpoints were ΔFVC and ΔFEV_1_ between the Spirofy™ devices 1 and 2 to test the inter-device variability.

### Statistical methods

Data on the baseline characteristics and other test parameters were summarized using descriptive statistics. Continuous variables are described using mean ± SD and minimum and maximum values; categorical variables are described using percentages and frequencies. The Kolmogorov–Smirnov (K–S) test was used to check if the data were normally distributed before data analysis was performed. The mean differences in spirometry measures between the Spirofy™ and Vitalograph™ Alpha Touch devices were analyzed with the Wilcoxon test or paired sample t-test depending upon the distribution of the data. The correlation coefficient was calculated to test if the measurements of both devices correlated with each other.

The coefficient of variation between two measurements obtained by the Spirofy™ was used to evaluate reproducibility (coefficient of variation = SD/mean) and the 95% limits of agreement (1.96*SD). The limits of agreement were calculated as mean difference ± 1.96*SD and Bland–Altman plots were created. The Bland–Altman method was used to validate if any systematic differences were present in the flow profile measurements taken from Spirofy™ 1 and 2. For comparison, the acceptable limits of agreement were considered as 0.50 L for FVC and 0.35 L for FEV_1_. There were no indeterminate test results or missing data from index or reference standard tests. No exploratory analysis was carried out. All statistical analyses were carried out using the Statistical Packages for Social Science (SPSS) version 27.0.

## Results

### Study population distribution

**Fig. 3:**
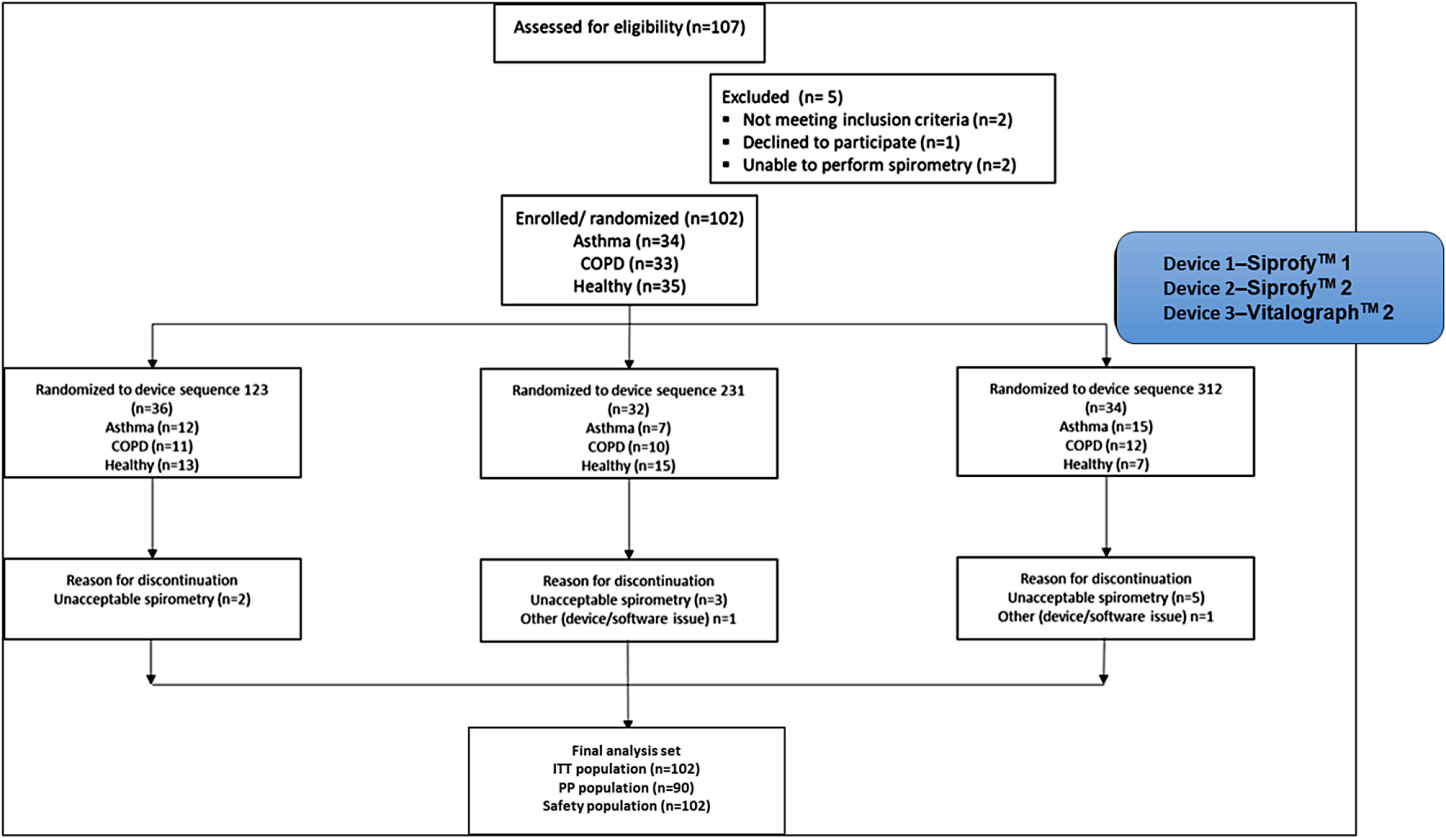
Consolidated Standards of Reporting Trials (CONSORT) diagram depicting study population distribution. COPD: Chronic obstructive pulmonary disease; ITT: Intention to treat; PP: Per protocol

The study enrolled 102 subjects randomly from a total of 107 subjects, with 35 healthy subjects, 34 subjects with asthma, and 33 subjects with COPD; these 107 subjects constituted the intention-to-treat (ITT) population. The enrolled subjects were randomized to device sequences 123, 231, or 312, where device 1 represented Spirofy™ 1, device 2 represented Spirofy™ 2, and device 3 represented the Vitalograph Alpha Touch™. Ninety subjects with acceptable spirometry test results, per protocol (PP) population, completed the study; these included 29 healthy subjects, 31 subjects with asthma, and 30 subjects with COPD (Fig. 3). The distribution of severity of the target condition is provided as supplementary information (SI-1). There were no alternate diagnoses in subjects without the target condition.

### Study population demographic characteristics

Among the 102 Indian subjects enrolled in the study and analyzed for the ITT population, 65.7% were men. The subjects had a mean age (± SD) of 49.8 (± 17.0) years. Of the 90 subjects analyzed for the PP population, 63% were men and the subjects had an average age (± SD) of 50.4 (± 16.7) years. Amongst the enrolled subjects, 35.6% reported chronic respiratory symptoms, 11% had a history of allergic rhinitis, and 24% had been exposed to tobacco smoke in the past. For subjects with asthma, the mean (± SD) disease duration was 4.19 (± 3.36) years; for subjects with COPD, this value was 6.04 (± 4.37) years (Table 1).

**Table 1:** Demographic characteristics of intention-to-treat and per-protocol population

Intention-to-treat population				
Characteristics (unit); statistics	Healthy (*N* = 35)	Asthma (*N* = 34)	COPD (*N* = 33)	Overall (*N* = 102)
Age (years); mean (SD)	34.2 (11.3)	53.0 (13.2)	63.03 (11.9)	49.8 (17.0)
Gender; n (%)				
Men	24 (68.6%)	13 (38.2%)	30 (90.9%)	67 (65.7%)
Women	11 (31.4%)	21 (61.8%)	3 (9.1%)	35 (34.3%)
**Per-protocol population**				
**Characteristics (unit); statistics**	**Healthy** (***N* = 29)**	**Asthma** ***N*** **( = 31)**	**COPD** (***N* = 30)**	**Overall** (***N* = 90)**
Age (years); mean (SD)	35.3 (11.4)	51.5 (12.6)	63.8 (12.2)	50.4 (16.7)
Gender; n (%)				
Men	19 (65.5%)	11 (35.5%)	27 (90.0%)	57 (63.3%)
Women	10 (34.5%)	20 (64.5%)	3 (10.0%)	33 (36.7%)
**Medical history**				
Symptoms (yes); n (%)	0 (0.0%)	13 (41.9%)	19 (63.3%)	32 (35.6%)
History of allergic rhinitis (yes); n (%)	0 (0.0%)	8 (25.8%)	2 (6.7%)	10 (11.1%)
History of exposure to tobacco smoke (yes); n (%)	1 (3.4%)	3 (9.7%)	18 (60.0%)	22 (24.4%)
Disease duration for asthma/COPD (years); mean (SD)		4.19 (3.36)	6.04 (4.37)	

COPD: chronic obstructive pulmonary disease; %: Percentage of subjects calculated relative to the total number of randomized subjects; N: Total number of randomized subjects; n: Number of subjects; SD: Standard deviation

### Medication use among the study population

The 31 asthma patients who completed the study were on inhaled corticosteroid (ICS)/long-acting beta-agonist (LABA) (28), ICS/LABA/long-acting muscarinic antagonist (LAMA) (1),xanthine derivatives (6), leukotriene receptor antagonist (LTRA) (6), antihistamine (5), short-acting muscarinic antagonist (SAMA)/short-acting beta-agonist (SABA) (1), oral corticosteroids (2), and N-acetylcysteine (1). The 30 COPD patients were on ICS/LABA (9), ICS/LABA/LAMA (14), LABA/LAMA (5), LAMA (1), ICS/LABA + LABA/LAMA (2), xanthine derivatives (18), LTRA (4), SAMA/SABA (1), oral corticosteroids (2), N-acetylcysteine (1). A list of all medications used by the asthma and COPD patient groups is provided in the supplementary information (SI-2).

### Primary endpoints

The mean ± SD FVC values reported for the Spirofy™ 1, Spirofy™ 2, and Vitalograph Alpha Touch™ were 2.60 ± 1.05, 2.64 ± 1.04, and 2.67 ± 1.04 L, respectively **(**Table 2**)**. The mean differences in FVC values between the Vitalograph Alpha Touch™ and Spirofy™ 1 were 71.44, 75.17, 27.74, and 113 mL for overall, healthy, asthma, and COPD groups, respectively **(**Table 3**)**. The mean differences in FVC values between the Vitalograph Alpha Touch™ and Spirofy™ 2 for the overall, healthy, asthma, and COPD groups were 35.89, 72.76, -15.16, and 53 mL, respectively (Table 3).

**Table 2:** Mean values of FVC and FEV_1_ in the per-protocol population as measured by the Spirofy™ 1, Spirofy™ 2, and Vitalograph Alpha Touch™ devices

Parameter/Device	Statistics	Healthy (*n* = 29)	Asthma (*n* = 31)	COPD (*n* = 30)	Overall (*n* = 90)
**FVC (L)**					
Spirofy™ 1	Mean (SD)	3.48 (1.03)	2.21 (0.83)	2.16 (0.68)	2.60 (1.05)
Spirofy™ 2	Mean (SD)	3.48 (1.05)	2.25 (0.86)	2.22 (0.65)	2.64 (1.04)
Vitalograph Alpha Touch™	Mean (SD)	3.56 (1.00)	2.24 (0.86)	2.27 (0.67)	2.67 (1.04)
**FEV**_**1**_ **(L)**					
Spirofy™ 1	Mean (SD)	2.75 (0.77)	1.55 (0.67)	1.33 (0.59)	1.87 (0.91)
Spirofy™ 2	Mean (SD)	2.76 (0.79)	1.58 (0.68)	1.35 (0.58)	1.88 (0.92)
Vitalograph Alpha Touch™	Mean (SD)	2.84 (0.76)	1.61 (0.69)	1.39 (0.60)	1.93 (0.93)

COPD: Chronic obstructive pulmonary disease; FEV_1_: Forced expiratory volume in one second; FVC: Forced vital capacity; n: Number of subjects in the analysis set; SD: Standard deviation

**Table 3:** The comparative limits of agreement and correlation coefficients for FVC and FEV_1_ in the per-protocol population measured by Spirofy™ 1, Spirofy™ 2, and Vitalograph Alpha Touch™ devices

Parameter/Device	Statistics	Healthy (*n* = 29)	Asthma (*n* = 31)	COPD (*n* = 30)	Overall (*n* = 90)
**FVC**					
Vitalograph Alpha Touch™ vs. Spirofy™ 1	Correlation coefficient	0.906^#^	0.963*	0.905^#^	0.963^#^
	Mean difference	75.17	27.74	113	71.44
	95% limit of agreement	(–645.9, 796.2)	(–426.4, 481.9)	(–234.3, 460.3)	(–455.0, 597.9)
Vitalograph Alpha Touch™ vs. Spirofy™ 2	Correlation coefficient	0.899^#^	0.972*	0.875^#^	0.955^#^
	Mean difference	72.76	–15.16	53	35.89
	95% limit of agreement	(–672, 817.5)	(–417.4, 387)	(–296, 402)	(–487.8, 559.6)
Spirofy™ 1 vs. Spirofy™ 2	Correlation coefficient	0.981*	0.990*	0.955^#^	0.986^#^
	Mean difference	–2.41	–42.90	–60.0	–35.6
	95% limit of agreement	(–272.9, 268.1)	(–289.9, 204.1)	(–321.6, 201.6)	(–295.5, 225.4)
**FEV**_**1**_					
Vitalograph Alpha Touch™ vs. Spirofy™ 1	Correlation coefficient	0.931*	0.974*	0.964^#^	0.981^#^
	Mean difference	83.10	58.39	55.67	65.44
	95% limit of agreement	(–400.1, 566.3)	(–248.7, 365.4)	(–110.9, 222.3)	(–273.5, 404.4)
Vitalograph Alpha Touch™ vs. Spirofy™ 2	Correlation coefficient	0.916*	0.974*	0.964^#^	0.977^#^
	Mean difference	72.76	31.61	37.0	46.67
	95% limit of agreement	(–559.6, 705.1)	(–275.7, 338.9)	(–147.0, 221.0)	(–365.6, 458.9)
Spirofy™ 1 vs. Spirofy™ 2	Correlation coefficient	0.982*	0.991*	0.981^#^	0.991^#^
	Mean difference	–10.34	–26.77	–18.67	–18.8
	95% limit of agreement	(–301.0, 280.4)	(–209.3, 155.7)	(–191.4, 154.0)	(–237.2, 199.6)

N: Number of subjects in the analysis set

*Correlation coefficient has been calculated using Pearson correlation statistics (normal data)

#Correlation coefficient has been calculated using Spearman’s correlation statistic (non-normal data)

95% limit of agreement = (mean difference − 1.96 SD, mean difference + 1.96 SD)

Strong positive correlations in the FEV_1_ values measured by the Vitalograph Alpha Touch™, Spirofy™ 1, and Spirofy™ 2 devices for the overall, healthy, asthma, and COPD groups were detected (Table 3). The mean ± SD FEV_1_ values reported for the Spirofy™ 1, Spirofy™ 2, and Vitalograph Alpha Touch™ were 1.87 ± 0.92, 1.88 ± 0.92, and 1.93 ± 0.93 L, respectively. The mean differences in FEV_1_ values between Vitalograph Alpha Touch™ and Spirofy™ 1 were 65.44 mL, 83.10 mL, 58.39 mL, and 55.67 mL for overall, healthy, asthma, and COPD groups, respectively (Table 3). The mean difference in FEV_1_ values between the Vitalograph Alpha Touch™ and Spirofy™ 2 for the overall, healthy, asthma, and COPD groups were 46.67, 72.76, 31.61, and 37.0 mL, respectively. (Table 3).

### Comparison of limits of agreement

**Fig. 4:**
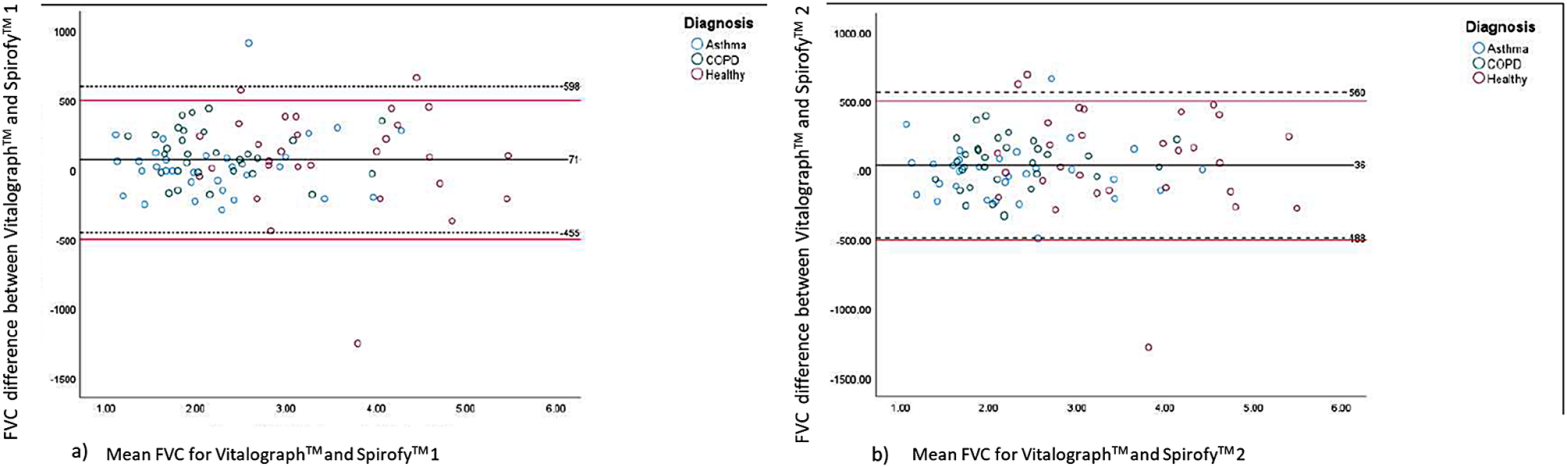
Bland-Altman plot for FVC overall population **a**)Vitalograph™ vs. Spirofy™ 1 and **b**) Vitalograph™ vs.Spirofy™ 2 COPD: Chronic obstructive pulmonary disease; FVC: Forced vital capacity

**Fig. 5:**
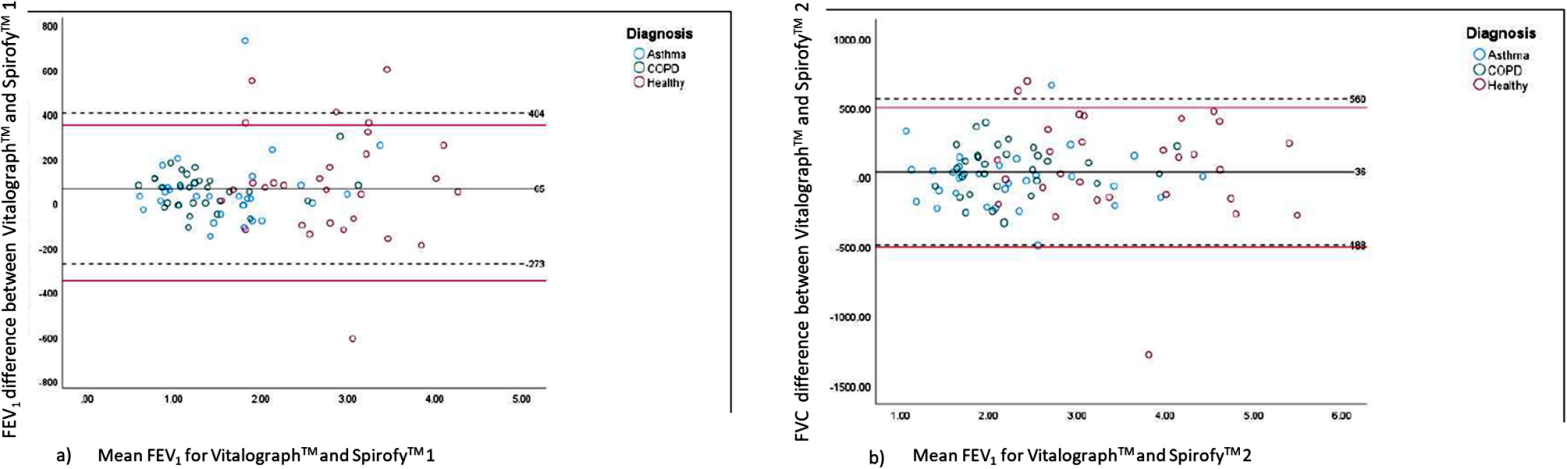
Bland-Altman plot for FEV_1_ overall population (**a**) Vitalograph™ vs. Spirofy™ 1 and (**b**) Vitalograph™ vs. Spirofy™ 2. COPD: Chronic obstructive pulmonary disease; FEV_1_: Forced expiratory volume in one second

The FVC values measured by Spirofy™ 1 and Spirofy™ 2 were within the pre-defined limits of agreement of those measured by the Vitalograph Alpha Touch™. The 95% FVC values for Spirofy™ 1 and Vitalograph Alpha Touch™ and those for Spirofy™ 2 and Vitalograph Alpha Touch™ were within the ± 500 mL range [14]. For the differences between the FEV_1_ values measured by Spirofy™ 1 and Vitalograph Alpha Touch™, 92.2% of values were within the predefined limits of ± 350 mL; in the case of Spirofy™ 2 and Vitalograph Alpha Touch™, 91.1% of these values were within this limit (Figs. 4a and b and 5a and b) [14].

### Co-primary endpoint

Diagnostic validation of Spirofy™ 1 using the Vitalograph Alpha Touch™ as a reference at an FEV_1_/FVC ratio < 0.7 showed that Spirofy™ 1 had good sensitivity (97.2%), specificity (90.1%), and overall accuracy (93.3%) in distinguishing airway obstructions from non-airway obstructions in the PP population (which included the healthy, asthma, and COPD groups). Similarly, Spirofy™ 2 also had good scores in sensitivity (97.2%), specificity (88.9%), and overall accuracy (92.2%) (Table 4).

**Table 4:** Distribution of FEV_1_/FVC < 0.7 between Spirofy™ 1 and Vitalograph Alpha Touch™ and Spirofy™ 2 and Vitalograph Alpha Touch™ in the overall group of the per-protocol population

Obstruction by Spirofy™ 1^a^	Obstruction by Vitalograph Alpha Touch^TM,b^			Overall	
	Yes	No			
Yes	35	5		40	
No	1	49		50	
Overall	36	54		90	
Sensitivity (%)	97.2				
Specificity (%)	90.1				
Accuracy (%)	93.3				
**Obstruction by Spirofy™ 2** ^**c**^	**Obstruction by Vitalograph Alpha Touch** ^TM,**b**^				**Overall**
	Yes		No		
Yes	35		6		41
No	1		48		49
Overall	36		54		90
Sensitivity (%)	97.2				
Specificity (%)	88.9				
Accuracy (%)	92.2				

^a, b, c^Obstruction is defined as FEV_1_/FVC < 0.7.

Sensitivity, specificity, and overall accuracy were calculated using the following formulae:

Sensitivity = number of true positives (TP)/(number of true positives (TP) + number of false negatives

(FN)).

Specificity = number of true negatives (TN)/(number of true negatives (TN) + number of false positives

(FP)).

Accuracy = number of true positives (TP) + number of true negatives (TP + TN)/total number of subjects

(TP + TN + FN + FP)

### Secondary endpoints

#### FVC

All FVC (100%) values measured by Spirofy™ 1 and Spirofy™ 2 were within the pre-defined limits of agreement with the values of ΔFVC between Spirofy™ 1 and Spirofy™ 2 being within the ± 500 mL range.

#### FEV_1_

For the overall group, the mean difference in FEV_1_ values between Spirofy™ 1 and Spirofy™ 2 was 18.8 mL, with the 95% limits of agreement ranging from − 237.2 to 199.6 mL. A strong positive correlation was observed in the FEV_1_ values measured between the Spirofy™ 1 and Spirofy™ 2 devices in the overall group. For the differences between the FEV_1_ values measured by Spirofy™ 1 and Spirofy™ 2, 98.8% of values were within the pre-defined limits of ± 350 mL.

### Safety findings

No adverse events were reported from performing the index test or the reference standard test.

## Discussion

Despite being common, obstructive airway diseases such as COPD and asthma are frequently underdiagnosed in clinical practice [15]. Previous studies have reported underdiagnosed asthma in 19–73% of individuals [16]. According to the Global Asthma Network (GAN) study conducted across multiple centers in urban parts of India, 75–82% of patients who had wheezing and 68–70% of subjects with severe asthma had not been clinically diagnosed with asthma [17]. The Global Initiative for Chronic Obstructive Lung Disease summit on COPD estimated that 95–98% of COPD cases are underdiagnosed in India as symptomatic patients seek healthcare advice late due to a lack of awareness; in addition, most COPD cases are diagnosed based on symptoms rather than spirometry [18].

Spirometry is an important and highly recommended test for the diagnosis of OAD [12]. It also helps in evaluating the severity of the disease and monitoring airflow obstruction [8]. Spirometry measurements obtained in childhood, adolescence, and adulthood can identify individuals at risk for non-communicable respiratory and non-respiratory diseases [19]. Despite these benefits, spirometry is still underutilized in clinical settings [12]. The main causes of this underutilization include the lack of precise, portable, and reasonably priced spirometers, particularly in primary care settings; difficulties in maintaining such equipment; and difficulties in conducting and interpreting spirometry tests [12, 14]. Most modern spirometers that are manufactured and sold globally are accurate and several are portable; however, they are expensive [20–22]. To address the rising burden of OAD in India, an indigenously manufactured, easy to use, accurate, inexpensive, and portable spirometer is critical.

In this study, we examined the clinical validity and accuracy of spirometry measurements obtained using the newly developed Spirofy™ spirometer, and compared it with the traditional Vitalograph Alpha Touch™ spirometer. Spirofy™ is a portable, pneumotach flow sensor-based spirometer with software designed with the capability for continuous improvements. The process to upgrade the device with the latest ATS/ERS algorithm has been initiated and will be completed within a year. A total of 90 participants were enrolled, including healthy individuals as well as people with asthma and COPD. The spirometry tests were performed at random with two Spirofy™ test devices (Spirofy™ 1 and Spirofy™ 2) and one Vitalograph Alpha Touch™ comparator device. This study demonstrates that the primary endpoint was attained by both test devices (Spirofy™ 1 and Spirofy™ 2), with > 95% of FVC and > 91% of FEV_1_ values falling within the acceptable limits of agreement. The limits of agreement for FVC and FEV_1_ values (±500 mL and ±350 mL) have been described previously for comparing two different types of spirometers [14]. This result is consistent with the findings of a case–control study that evaluated the accuracy of the Vitalograph® lung monitor and compared it to post-bronchodilator confirmatory spirometry tests used to screen COPD patients; the study revealed that the FEV_1_ values from the two devices were strongly correlated (*r* = 0.97) [22]. Another study that compared a portable spirometer (AioCare®) with a reference desktop spirometer demonstrated good correlation between the two devices in measuring peak expiratory flow readings, FEV_1_, FVC, and FEV_1_/FVC in patients with asthma or COPD [23]. A meta-analysis of 31 studies has shown that portable spirometers are highly accurate in diagnosing COPD [24]. Among the three most widely used portable spirometers, PIKO-6 was the most accurate, followed by COPD-6 and peak expiratory flow [24]. Conversely, portable turbine spirometers (CareFusion, Yorba Linda) and desktop laboratory spirometers (Vmax Encore System) were found to be uninterchangeable for use in children for diagnosing/monitoring cystic fibrosis owing to repeatability discrepancies in FEV_1_ values [25]. Furthermore, as Carpenter et al. (2018) have pointed out in their review, differences exist in the accuracy and quality of the assessment of lung function with portable spirometers, and not all are equally accurate [26].

Portable spirometers are utilized for lung function measurements in community settings and for tracking lung function over time in epidemiological studies. However, in large multicenter studies, different spirometers may be used across study sites and old models may need to be substituted with newer ones while a study is being conducted. This makes it necessary that the variability between different portable spirometers be determined so that the data collected across different devices are comparable [27]. In our study, all FVC values recorded by the Spirofy™ 1 and Spirofy™ 2 were within the pre-defined levels of agreement with the ΔFVC values lying within the ± 500 mL range. For the ΔFEV_1_ values measured by Spirofy™ 1 and Spirofy™ 2, 98.8% of the values were within the predefined limits of ± 350 mL. This indicates that both test devices meet the secondary endpoint and that there is no device-specific heterogeneity between the two test devices.

Another important objective of this study was to assess whether the Spirofy™ test devices were as good as the Vitalograph Alpha Touch™ in their ability to identify airway obstructions. Both the Spirofy™ devices exhibited high sensitivity (97%), specificity (about 90%), and overall accuracy (∽ 93.3%) in the diagnosis of and differentiation between different types of airway obstructions in obstructive pulmonary diseases. From the sensitivity and specificity exhibited by the Spirofy™ devices, it can be inferred that these devices successfully identified airflow obstruction in 97% of cases and failed to do so in 3% of the cases using a fixed cut-off value of 0.7 for the FEV_1_/FVC ratio. Compared to the Vitalograph Alpha Touch™, the Spirofy™ devices over-identified airway obstruction in approximately 10% of cases. These findings suggest that the Spirofy™ device is as effective as the Vitalograph Alpha Touch™ device in diagnosing airway obstructions in clinical settings. A similar efficacy has been observed in another study involving spirometers using an FEV_1_/FVC ratio < 0.7 in detecting obstruction; in this study, the Air Smart Spirometer was found to have a sensitivity of 90.4% and a specificity of 97.2% [15].

For people with asthma and COPD, the FVC values measured by Spirofy™ 1 and Spirofy™ 2 were within the pre-defined limits of agreement with those measured by the Vitalograph Alpha Touch™. Similarly, the FEV_1_ values measured by all three devices for people with COPD and asthma were also within the 95% limits of agreement. Therefore, the Spirofy™ was as capable as the Vitalograph Alpha Touch™ in identifying people with asthma and COPD.

Overall, the Spirofy™ is a portable, easy-to-use, pneumotach sensor-based spirometer that is as capable as the conventional Vitalograph Alpha Touch™ spirometer in detecting airway obstruction in patients with asthma and COPD. This device, which provides reliable and reproducible results, can also be a useful and effective diagnostic tool for OAD when combined with clinical history and examination results. This may improve accessibility for respiratory disease diagnoses in primary care settings.

The main strength of this study was its open-label, randomized, cross-over design. The open-label design was deemed suitable because the spirometry variables were objectively measured results that are unlikely to be influenced by subject or investigator bias. The order of use of the spirometers was randomized to ensure that the usage sequences of the devices were balanced within the study population. This study provides the most accurate and unbiased estimations for the differences in performance across devices by randomly assigning the subjects to each of the three device sequences. The cross-over design, in which each subject acts as a control for his/her tests, reduced the effects of potential confounders like intra-subject variability.

While the study provides valuable insights into the performance and clinical utility of Spirofy™, there are some limitations that need to be taken into consideration to contextualize the findings and guide further research. The number of participants were limited and the demographic was specific to India, which may restrict the generalizability of findings across different global populations. Assessment of operational variables, including ease of use and performance in the hands of individuals with varying levels of expertise, will provide insights into the wide applicability of the device. These limitations highlight the need for further research, including larger-scale, diverse population studies, and long-term reliability assessments to fully understand Spirofy™’s utility and applicability in broader clinical and non-clinical settings.

## Conclusion

This study demonstrates that Spirofy™, an easy-to-use and portable spirometer, is as accurate as the gold standard Vitalograph Alpha Touch™ spirometer for detecting airflow obstruction, with a sensitivity of 97%, specificity of 90%, and overall accuracy of 93.3%. Spirofy™ can thus be an alternative spirometer that can be used in clinical settings for the diagnosis of OAD.

### Electronic supplementary material

Below is the link to the electronic supplementary material.

Supplementary Material 1

Supplementary Material 2

Supplementary Material 3

## Data Availability

The datasets used and/or analyzed during the current study are available from the corresponding author upon reasonable request.

## Abbreviations

ATS/ERS: American Thoracic Society/European Respiratory Society
CI: Confidence interval
CONSORT: Consolidated Standards of Reporting Trials
COPD: Chronic obstructive pulmonary disease
COVID: Coronavirus disease
FVC: Forced vital capacity
FEV_1_: Forced expiratory volume
GAN: Global Asthma Network
IEC/IRB: Independent Ethics Committee/Institutional Review Board
ITT: Intention to Treat
K-S: Kolmogorov–Smirnov
N: Total number of randomized subjects
OAD: Obstructive airway diseases
PIS: Patient information sheet
PP: Per protocol
%: Percentage of subjects calculated relative to the total number of randomized subjects
QA: Quality assurance
RT-PCR: Real-time-polymerase chain reaction
SARS-CoV-2: Severe acute respiratory syndrome coronavirus
SD: Standard deviation
SPSS: Statistical Packages for Social Science
