# Supplementary material for: Comparison of a portable, pneumotach flow-sensor–based spirometer (Spirofy™) with the vitalograph alpha Touch™ spirometer in evaluating lung function in healthy individuals, asthmatics, and COPD patients—a randomized, crossover study
Source: BMC Pulm Med. 2024 May 10;24:230. doi: 10.1186/s12890-024-02972-4 (PMC11088097; doi:10.1186/s12890-024-02972-4)
Supplement: Supplementary file 1 — Supplementary Material 1 [file 12890_2024_2972_MOESM1_ESM.docx]

**Supplementary Information 2 (SI-2):** Medication use among study participants

Asthma (N=31)

| **Pharmacotherapy** | **Number of Patients** | **%** |
| --- | --- | --- |
|  |  |  |
| ICS/LABA | 28 | 90.32 |
| ICS/LABA/LAMA | 1 | 3.22 |
| Xanthine Derivatives | 6 | 19.35 |
| LTRA | 6 | 19.35 |
| Antihistaminic | 5 | 16.12 |
| SAMA/SABA | 1 | 3.22 |
| Oral Corticosteroids | 2 | 6.45 |
| N-Acetylcysteine | 1 | 3.22 |

ICS/LABA = Formoterol/Budesonide, Salmeterol/Fluticasone, Formoterol/Fluticasone

LTRA (Leukotriene Receptor Antagonist) = Montelukast

Oral Bronchodilators = Acebrophylline, Doxophylline, Theophylline

Antihistaminic = Levocetrizine

SAMA/SABA = Ipratropium/Salbutamol

COPD (N=30)

| **Pharmacotherapy** | **Number of Patients** | **%** |
| --- | --- | --- |
|  |  |  |
| ICS/LABA | 9 | 30 |
| ICS/LABA/LAMA | 14 | 46.66 |
| LABA/LAMA | 5 | 16.66 |
| LAMA | 1 | 3.33 |
| ICS/LABA + LABA/LAMA | 2 | 6.66 |
| Xanthine Derivatives | 18 | 60 |
| LTRA | 4 | 13.33 |
| SAMA/SABA | 1 | 3.33 |
| Oral Corticosteroids | 2 | 6.66 |
| N-Acetylcysteine | 1 | 3.33 |

LAMA – glycopyrronium

ICS/LABA – Formoterol/Budesonide, Salmeterol/Fluticasone

ICS/LABA/LAMA – Budesonide/Glycopyrronium/Formoterol; Ciclesonide/Tiotropium/Formoterol; Salmeterol/Fluticasone/Tiotropium

LABA/LAMA – formoerol/glycopyrronium, indacaterol/glycopyrronium

Co-ICS/LABA + LABA/LAMA – Formoterol/Budesonide + Indacaterol/Glycopyrronium

Oral Bronchodilators – Acebrophylline, Doxophylline, Theophylline

Leukotriene Receptor Antagonists – montelukast

SAMA/SABA – Ipratropium/Salbutamol
