# Supplementary material for: Comparison of a portable, pneumotach flow-sensor–based spirometer (Spirofy™) with the vitalograph alpha Touch™ spirometer in evaluating lung function in healthy individuals, asthmatics, and COPD patients—a randomized, crossover study
Source: BMC Pulm Med. 2024 May 10;24:230. doi: 10.1186/s12890-024-02972-4 (PMC11088097; doi:10.1186/s12890-024-02972-4)
Supplement: Supplementary file 2 — Supplementary Material 2 [file 12890_2024_2972_MOESM2_ESM.docx]

**SI-1: Disease severity distribution (based on Vitalograph Alpha Touch^TM^– the reference standard)**

1. Criteria for severity grading

| Severity | Asthma | COPD |
| --- | --- | --- |
| Mild | FEV_1_ >80% predicted | FEV_1_ >80% predicted |
| Moderate | 60< FEV_1_ % Predicted <80 | 50 <FEV_1_ % Predicted <80 |
| Severe | FEV_1_ <60% predicted | 30 <FEV_1_ % Predicted <50 |
| Very severe |  | FEV_1_ <30% predicted |

1. The severity distribution for target conditions is summarized in the following table:

| Disease Severity | Asthma | | COPD | |
| --- | --- | --- | --- | --- |
| Mild | 14 | 45% | 4 | 13% |
| Moderate | 7 | 23% | 17 | 57% |
| Severe-Very Severe | 10 | 32% | 9 | 30% |
|  | 31 |  | 30 |  |
